# Characterization of Atherosclerotic Plaque in Coronary CT Angiography and Some Related Factors in Patients with Coronary Artery Disease Referred to Farshchian Heart Hospital in Hamadan in 2023 -

**DOI:** 10.31661/gmj.vi.3702

**Published:** 2025-05-25

**Authors:** Zahra Hamian, Seyed Kamal Hadei, Zahra Khanlarzadeh, Farnaz Fariba

**Affiliations:** ^1^ Hamadan University of Medical Sciences, Hamadan, Iran

**Keywords:** Coronary Vessels, Atherosclerotic Plaque, Coronary Angiography

## Abstract

**Background:**

Vascular classification in coronary artery disease is influenced by atherosclerotic plaque characteristics. This study aimed to investigate the characterization of atherosclerotic plaque and some related factors in coronary CT angiography in patients with coronary artery disease referred to Farshchian Heart Hospital in Hamadan in 2023

**Materials and Methods:**

In this analytical cross-sectional study that was conducted in 2023 in Hamadan, Iran, 140 individuals suspected of coronary artery stenosis based on atherosclerotic plaque characteristics in coronary angiography were examined. The study analyzed the relationship between plaque features, demographic characteristics, degree of coronary artery stenosis, and other risk factors for coronary artery disease with chi-square, logistic regression, correlation coefficient using SPSS version 20.

**Results:**

The mean age of patients was 53.11±7.62 years, with 59% male and 40% female. Among patients, 40% had coronary artery stenosis, with 18% having severe stenosis. The prevalence of positive remodeling was 32%, Low Attenuation was 45%, Napkin-ring Sign was 14%, and Spotty Calcium was 25%. Significant associations were found between various plaque patterns and age (P0.05), Low Attenuation pattern with hypertension (P0.001), diabetes (P=0.03), dyslipidemia (P=0.04), Napkin-ring Sign with diabetes (P=0.03).

**Conclusion:**

The study highlights the high prevalence of distinct plaque patterns and their associations with severity of coronary artery stenosis, presence of diabetes, hypertension, and dyslipidemia. These findings emphasize the need for tailored risk assessment and management strategies in patients with coronary artery disease.

## Introduction

The heart, as the main body organ, needs oxygen and nutrients supplied by the coronary arteries. Blockage or narrowing of these arteries can lead to heart failure and heart attacks [1]. In 2015, cardiovascular diseases (CVDs) led to 17.70 million global deaths, representing 31% of total deaths. CVDs encompass coronary artery disease, strokes, and rheumatic heart disease. Coronary artery disease and stroke were responsible for 7.40 million and 6.70 million deaths, respectively. Three-quarters of CVD deaths occur in low- and middle-income countries, with 82% of premature non-communicable disease deaths in these regions attributed to CVDs [2][3][4].

Modifiable risk factors for CAD include high blood pressure, high cholesterol, smoking, diabetes, obesity, low physical activity, unhealthy diet, and stress. Over the past few decades, advancements in cardiovascular care have reduced mortality rates [5].

Invasive coronary angiography has traditionally been used to diagnose CAD; however, coronary computed tomography angiography (CCTA) has emerged as a non-invasive alternative. This test is increasingly used as a first-line diagnostic modality, allowing for personalized medical care and interventions based on plaque type [6].

Plaque examinations show three key features linked to heart attacks: rupture, erosion, and calcified nodules. Most thrombi in heart attacks result from ruptured atherosclerotic lesions with thin fibrous caps covering necrotic cores [7]. The concept of plaque vulnerability, specifically thin-cap fibroatheromas (TCFA), with a cap thickness of <65 µm, is linked to plaque rupture. Current CT scanners have limited resolution for analyzing fibrous caps; nevertheless, CCTA can help detect vulnerable plaques by assessing plaque composition [8].

While CAD is a global health concern, regional variations in its prevalence, risk factors, and plaque characteristics are significant. Our study is the first to provide detailed insights into atherosclerotic plaque characteristics among patients in Hamadan, Iran. This localized focus addresses a gap in the literature, as previous studies have predominantly centered on other regions. We utilized coronary CTA to non-invasively detect, characterize, and quantify atherosclerotic plaques. This approach aligns with recent advancements in imaging modalities that enhance the assessment of coronary atherosclerosis [9]. By applying these advanced techniques in our study, we contribute to the growing body of evidence supporting their clinical utility. Furthermore, by providing this level of detail, our study enhances the understanding of plaque vulnerability in the context of CAD [10].

This study introduces novel insights into the characterization of atherosclerotic plaques in a previously underrepresented population, utilizing advanced imaging techniques to provide comprehensive data that can enhance regional healthcare strategies.

Given the importance of diagnosing high-risk plaques for determining appropriate treatment plans, the present study was conducted to examine the characterization of atherosclerotic plaque and some related factors in coronary CT angiography in patients with CAD referred to Farshchian Heart Hospital in Hamadan in 2023.

## Materials and Methods

### 1.Study Design and Setting

The present analytical cross-sectional study was conducted using an observational analytical research design. It aimed at examining the characteristics and associations of atherosclerotic plaques in coronary angiography in

### 2.Study Participants/ Population

This study was conducted on patients who referred to Farshchian Heart Hospital in Hamedan in 2023 and underwent coronary CT angiography (CCTA). Farshchian Heart Hospital is one of the specialized heart centers in Hamedan province, which is known as a reference for the diagnosis and treatment of cardiovascular diseases. The center accepts a wide range of patients from all over the province and even neighboring cities from other provinces. The majority of patients who refer are middle-aged and elderly, as coronary artery disease (CAD) is usually more common in older ages. Many patients have cardiovascular risk factors such as hypertension, diabetes, hyperlipidemia, family history of heart disease, and smoking. Patients who refer to this center mainly present with symptoms such as chest pain, shortness of breath, decreased exercise tolerance, and symptoms related to myocardial ischemia. This hospital acts as a referral center, so some patients are referred to this center from other medical centers and clinics in the city and province of Hamedan. Some patients have a history of other diagnostic tests such as exercise testing, echocardiography, and invasive angiography, which are considered as criteria for deciding whether to perform CCTA. The socioeconomic level of the patients is diverse, but the majority of the clients are middle-class and retired.

### 3.Study Variables and Definition

### 3.1.CTA Protocol

All patients suspected of having CAD who did not undergo immediate treatment (such as percutaneous coronary intervention) and were candidates for coronary angiography were included in the study after obtaining their informed consent. Before coronary angiography, patients were prepared for the procedure involving blood urea nitrogen test, creatinine tests, fasting, and heart rate adjustment between 60-70. After obtaining appropriate vein access on the left hand, injection was performed with proper angiocath and nitroglycerin spray. Plaque characterization in CCTA was performed using a standardized protocol. Images were taken using a Siemens 128-slice CT angiography machine with a slice thickness of 0.60 mm and a temporal resolution of 75 ms and tube voltage of 128 kVp. Plaque analysis was conducted using Syngo. Plaques were categorized into Positive remodeling, Low attenuation, Napkin-ring Sign and Spotty calcium. All images were analyzed by two independent radiologists with over 10 years of experience, and discrepancies were resolved by consensus

Sociodemographic characteristics of the patients; age (years), gender (male/female), Family history of coronary artery disease (yes/no), body mass index (BMI; kh/m2) and related heart disorders including High blood pressure (yes/no), Diabetes (yes/no), Dys lipidemia (yes/no), Degree of coronary artery stenosis (Stenosis (+), Stenosis (-) and Sever stenosis) were entered into a checklist. Patients undergoing coronary angiography were evaluated for plaque results by a cardiac imaging fellowship and recorded on the checklist. They are categorized into Positive remodeling, Low attenuation, Napkin-ring Sign and Spotty calcium

### 4.Sample Size Consideration

Considering that this study aimed to compare the quantitative and qualitative characteristics of plaques in patients with low, moderate, and high risk based on a medium effect size of 0.13 (Cohen’s effect size>0.13), as defined by Cohen’s guidelines, where a medium effect size represents a moderate degree of difference between groups, a type I error of 5%, and a test power of 80%, a sample size of 132 individuals was determined. However, the study involved the examination of 140 individuals.

### 5.Ethical Statement

This study was conducted in accordance with the principles outlined in the Declaration of Helsinki. Ethical approval was obtained from the Institutional Review Board (IRB) of Farshchian Heart Hospital, Hamadan (approval number: IR.UMSHA.REC.1402.421). All participants provided written informed consent after receiving detailed explanations about the study’s purpose, procedures, and potential risks. Confidentiality was maintained by anonymizing patient data and restricting access to study records.

### 6.Eligibility Criteria

Inclusion criteria for the study were suspicion of CAD, failure to undergo immediate treatment (including percutaneous coronary intervention), undergoing coronary angiography, and consent to participate in the study. On the other hand, exclusion criteria were lack of access to patient data and patient dissatisfaction with participating in the study.

### 7.Statistical Tests

The collected data were analyzed using SPSS version 20 software. Qualitative variables were described as frequencies and percentages, and quantitative variables were presented as mean ± Standard deviation (SD). In order to compare the characteristics of atherosclerosis plaques, the Student’s t-test was employed for they by age and body mass index (BMI), while the Chi-square test was used for they by gender, family history of coronary disease, and other variables, such as diabetes, hypertension, dyslipidemia, and smoking status. For the determination of correlation between coronary plaque burden and age, it is used Pearson correlation. In this study, the statistical significance level was considered at 5%. Furthermore, the odds ratios (ORs) and 95% confidence intervals (CIs) were calculated by use of unconditional logistic regression to assess the associations between the variables.

## Results

**Figure-1:**
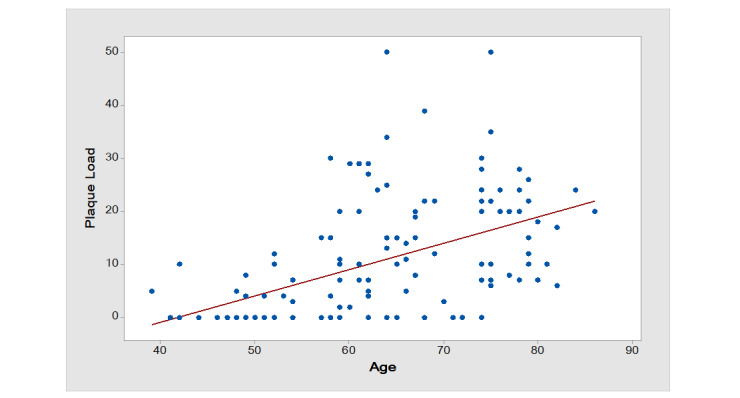
Figure 1. Distribution of coronary plaque burden according to age (line regression and R2 = 0.20)

**Table:** Table1. Frequency Distribution of Atherosclerotic Plaque Characteristics in CT Coronary Angiography of Patients with Coronary Artery Disease Referred to Farshchian Heart Hospital in Hamedan in 2023

**Atherosclerotic plaque characteristics**	**n**	**Percent (%)**
Positive remodeling		
(-) (+) Total	95 45 140	67.9 32.1 100
Low attenuation		
(-) (+) Total	76 64 140	54.3 45.7 100
Napkin-ring Sign		
(-) (+) Total	120 40 140	85.7 14.3 100
Spotty calcium		
(-) (+) Total	104 36 140	74.3 25.7 100

**Table:** Table2. Frequency Distribution of Atherosclerotic Plaque Characteristics in CT Coronary Angiography of Patients with Coronary Artery Disease Referred to Farshchian Heart Hospital in Hamedan in 2023 by Age and Family History of Coronary Artery Disease and Related Odds Rations and Confidence Intervals 95%.

**Atherosclerotic ** **plaque characteristics **	**Gender**		**OR (CI 95%), P-value **	**Family history of ** **coronary artery disease **		
	**Male**	**Female**		**No**	**Yes**	**OR (CI 95%), P-value**
Positive remodeling						
(-) (+)	57 26	38 19	0.91 (0.44, 1.87), 0.80	58 31	37 17	0.06 (0.04, 0.10), 0.37
Low attenuation						
(-) (+)	42 41	34 23	1.44 (0.73, 2.85), 0.29	45 44	31 20	0.63 (0.31, 1.27), 0.24
Napkin-ring Sign						
(-) (+)	68 15	52 5	2.29 (0.78, 6.72), 0.12	73 47	16 4	2.58 (0.81, 8.18), 0.10
Spotty calcium						
(-) (+)	61 22	43 14	1.11 (0.51, 2.41), 0.80	64 25	40 11	0.11 (0.05, 0.24), 0.40

**Table:** Table3. Frequency Distributions of Atherosclerotic Plaque Characteristics in Coronary CT Angiography of Patients with Coronary Artery Disease According to Hypertension, Diabetes, and Dyslipidemia and Related Odds Rations and Confidence Intervals 95%.

**Atherosclerotic ** **plaque characteristics **	**High blood pressure **		**OR (CI 95%), P-value**	**Diabetes**			**Dyslipidemia**		
	**No**	**Yes**		**No**	**Yes**	**OR (CI 95%), P-value **	**No**	**Yes**	**OR (CI 95%), P-value **
Positive remodeling									
(-) (+)	31 19	64 26	1.51 (0.73, 3.13), 0.27	74 30	21 15	0.57 (0.26, 1.25), 0.16	66 32	29 13	1.08 (0.5, 2.36), 0.83
Low attenuation									
(-) (+)	60 30	16 34	0.24 (0.11, 0.49), 0.001	64 40	12 24	0.31 (0.14, 0.69), 0.003	59 39	17 25	0.45 (0.22, 0.94), 0.03
Napkin-ring Sign									
(-) (+)	79 11	41 9	0.63 (0.24, 1.65), 0.35	93 11	27 9	0.35 (0.13, 0.95), 0.033	88 10	32 10	0.36 (0.14, 0.95), 0.04
Spotty calcium									
(-) (+)	67 23	37 13	0.98 (0.44, 2.15), 0.95	80 24	24 12	0.6 (0.26, 1.38), 0.26	75 23	29 13	0.68 (0.31, 1.53), 0.325

**Table:** Table4. Frequency Distributions of Atherosclerotic Plaque Characteristics in CT Coronary Angiography of Patients with Coronary Artery Disease According to the Degree of Coronary Artery Stenosis.

**Atherosclerotic ** **plaque characteristics **	**Degree of coronary artery stenosis**			P-value*
	**Stenosis (+)**	**Stenosis (-)**	**Sever stenosis**	
Positive remodeling				
(-) (+)	20 (40.8) 29 (59.2)	73 (84.9) 13 (15.1)	2 (0.4) 3 (0.6)	˂0.001
Low attenuation				
(-) (+)	10 (20.4) 39 (79.6)	65 (75.6) 21 (24.4)	1 (0.2) 4 (0.8)	˂0.001
Napkin-ring Sign				
(-) (+)	36 (73.5) 13 (26.5)	79 (91.9) 7 (8.1)	5 (100) 0 (0)	0.01
Spotty calcium				
(-) (+)	27 (55.1) 22 (44.9)	73 (84.9) 13 (15.1)	4 (0.8) 1 (0.2)	0.001

This study examined a total of 140 patients with CAD. The frequency distribution of atherosclerotic plaque characteristics (Table-1) in coronary angiography in patients with CAD was shown to be low attenuation (45.70%), positive remodeling (32.10%), spotty calcium (25.70%), and napkin-ring sign (14.30%).The mean age of the patients was 53.11±7.62 years (range 39-86 years), and the mean BMI was 30.24±2.74 (range 17-31 kg/m2). Regarding gender, males accounted for 83 individuals (59.30%) and females for 57 individuals (40.70%) (Table-2). There was no significant association between gender or family history of CAD and atherosclerotic plaque characteristics in coronary angiography. For positive remodeling, the odds for males compared to females were slightly lower (OR = 0.91, 95% CI: 0.44-1.87), while individuals with a family history showed significantly lower odds (OR = 0.06, 95% CI: 0.04-0.10). Low attenuation plaques had higher odds for males (OR = 1.44, 95% CI: 0.73-2.85), and those with a family history had reduced odds (OR = 0.63, 95% CI: 0.31-1.27), though these findings were not statistically significant. For napkin-ring sign, males had more than twice the odds compared to females (OR = 2.29, 95% CI: 0.78-6.72), and a family history was associated with higher odds (OR = 2.58, 95% CI: 0.81-8.18), though neither reached statistical significance. Spotty calcium showed similar odds between males and females (OR = 1.11, 95% CI: 0.51-2.41), while individuals with a family history had significantly lower odds (OR = 0.11, 95% CI: 0.05-0.24) (Table-2).

The prevalence of risk factors for CAD included family history of CAD (36%), hypertension (35%), dyslipidemia (30%), diabetes (25%), in descending order (Table-3). However, a significant difference was observed between low attenuation and hypertension, as well as between diabetes and low attenuation and napkin-ring sign. According to the Table-3, High blood pressure is significantly associated with lower odds of Low attenuation plaques (OR=0.24; CI: 0.11-0.49). Similarly, diabetes (OR=0.31; CI: 0.14-0.69) and dyslipidemia (OR=0.45; CI: 0.22-0.94) also showed reduced odds. For Napkin-ring sign: Diabetes (OR=0.35; CI: 0.13-0.95) and dyslipidemia (OR=0.36; CI: 0.14-0.95) were associated with significantly lower odds. Other associations were not statistically significant.Regarding coronary artery stenosis, 35% of the subjects had stenosis, 3.40% had severe stenosis, and 61.40% had no stenosis (Table-4). Table-4 provides information about the frequency of atherosclerotic plaque characteristics in coronary angiography in patients with CAD based on the degree of coronary artery stenosis. Significant differences were observed between the degree of coronary artery stenosis in terms of positive remodeling (P<0.001), low attenuation (P<0.001), napkin-ring sign (P=0.011), and spotty calcium (P<0.001).

Significant differences were observed in the characteristics of atherosclerotic plaques in coronary angiography regarding age but not IBM.

The mean coronary plaque burden in our study was 35.14 ± 9.31 (range: 0-50). This wide range reflects the diverse severity of coronary artery disease across the study population. Figure-1 demonstrates a positive correlation between plaque burden and patient age (Pearson correlation coefficient: [insert value, e.g., r=0.45, P<0.001]). Notably, while the mean plaque burden is relatively low, the upper limit of the range highlights a subset of patients with significant disease, which aligns with the inclusion of low-, moderate-, and high-risk individuals in the study.

## Discussion

This study highlights the prevalence and clinical significance of specific high-risk morphological features of atherosclerotic plaques in patients with coronary artery disease (CAD). The findings emphasize the predominance of low attenuation, positive remodeling, spotty calcium, and napkin-ring sign among the studied population. These features are well-recognized markers of plaque vulnerability, reflecting an elevated risk for adverse cardiovascular events.

Bittner et al. (2018) investigated patients suspected of acute coronary syndrome undergoing coronary angiography. They found that the prevalence of high-risk coronary plaques, including remodeling index, plaque burden, and attenuation level, was 54% in coronary angiography [11], which was consistent with our findings.

The observed associations between plaque characteristics and patient age underline the progressive nature of atherosclerosis. In this study, a significant association was observed between the characteristics of coronary atherosclerotic plaques in coronary angiography and the age of patients. Patients with atherosclerotic plaques showing attenuation, spotty calcium, and napkin-ring sign were significantly older on average compared to patients without atherosclerotic plaques. In line with our results, Kataoka et al. (2012) found that patients with spotty calcium in coronary arteries were significantly older [12]. These findings suggest that plaque morphology may provide valuable insights into patient risk stratification beyond traditional clinical parameters.The study also indicates the interplay between specific cardiovascular risk factors and high-risk plaque features. Finck et al. (2020) reported that in assessing the long-term predictive value of morphological features of coronary angioplasty plaques, morphological features, such as spotty or gross calcification patterns and napkin-ring sign, were predictors of non-fatal myocardial infarction events [13]. These results reinforce the importance of aggressive management of modifiable risk factors, particularly in individuals exhibiting high-risk plaque features.

In the current study, among the risk factors for CAD (i.e., family history of coronary disease, hypertension, diabetes, dyslipidemia, and smoking), a significant association was observed between hypertension and low attenuation, diabetes and low attenuation and napkin-ring sign, and dyslipidemia and napkin-ring sign. In the study by Kataoka et al. (2012), a significant association was found between spotty calcification and a history of diabetes, myocardial infarction, and lower levels of high-density lipoprotein cholesterol during treatment [12]. According to the results reported by Jadidi et al. (2021), creatinine levels, CAD, and high blood pressure were the most influential factors in coronary artery calcification. However, race, smoking, diabetes, dyslipidemia, alcohol consumption, and drug use had minimal impacts on calcification. Vascular morphometry had no direct and independent effect on calcium burden [14]. Moreover, in the present study, a significant association was observed between higher IBM and increased severity of coronary artery calcification. Consistent with our findings, Jadidi et al. (2021) found a significant association between IBM and coronary artery calcification [14]. Therefore, these findings suggests that obesity may independently contribute to atherosclerotic progression. This highlights the importance of incorporating BMI into risk assessments and interventions for patients with CAD, given its potential role in accelerating disease progression.

Overall, the findings of this study underscore the utility of coronary computed tomography angiography (CCTA) not only for anatomical imaging but also for identifying high-risk plaque characteristics that may guide individualized risk stratification and management. The significant associations observed between plaque morphology, patient demographics, and risk factors reinforce the need for a tailored approach to cardiovascular prevention and treatment.

This study has several limitations that should be considered when interpreting the results. First, the potential for selection bias exists as the study population was drawn from patients referred to a single center, which may limit the generalizability of the findings to broader populations [15][16]. Second, while CCTA is a valuable imaging modality for characterizing coronary plaques, it has known limitations, particularly in detecting small, non-calcified plaques or distinguishing between certain plaque compositions. The accuracy of plaque assessment is also dependent on image quality, which can be affected by factors such as heart rate and patient body mass index [17][18].

The sample size, while sufficient for statistical analysis, may not fully capture the variability of plaque burden and characteristics across diverse patient populations. Additionally, there is the potential for referral bias, as patients included in the study were those referred for advanced imaging, possibly reflecting a subset with more advanced or symptomatic disease [19].

This study is limited by its cross-sectional design, which precludes the ability to establish causal relationships between risk factors and atherosclerotic plaque characteristics.

Lastly, the study’s cross-sectional design precludes unmeasured confounders such as lifestyle factors or medication use could influence the observed associations [20].

One of the limitations of this study was related to the unwillingness of some patients to participate in the study. Identifying the influence of demographics and risk factors on the prevalence of calcium can help better understand the pathophysiology of the disease and early diagnosis of patients at higher risk of cardiovascular events.

Despite these limitations, the findings provide valuable insights into the relationship between coronary plaque characteristics and clinical risk stratification, underscoring the importance of comprehensive imaging in the management of coronary artery disease. Future studies with larger, more diverse populations are warranted to validate these findings and explore the longitudinal implications of plaque morphology on cardiovascular outcomes.

## Conclusion

This study underscores the significant role of coronary computed tomography angiography (CCTA) in characterizing high-risk morphological features of atherosclerotic plaques in patients with coronary artery disease (CAD). The findings reveal the predominance of low attenuation, positive remodeling, spotty calcium, and napkin-ring sign, all of which are markers of plaque vulnerability associated with increased cardiovascular risk. Moreover, the observed associations between plaque characteristics, patient age, and specific risk factors such as hypertension, diabetes, dyslipidemia, and body mass index (BMI) highlight the interplay between clinical risk factors and atherosclerotic progression. These results emphasize the need for tailored cardiovascular risk stratification and management strategies, integrating imaging-based plaque morphology assessments and traditional clinical parameters. Despite the study’s limitations, including a single-center design, potential referral bias, and cross-sectional methodology, the findings provide valuable insights into the utility of CCTA as a tool for risk assessment and personalized treatment planning. This knowledge can ultimately lead to advancements in early detective and diagnostic methods for individuals who are at a heightened risk of cardiovascular events.

Future studies with larger, more diverse populations are warranted to validate these findings and explore the longitudinal implications of plaque morphology on cardiovascular outcomes.

## Conflict of Interest

None declared.
